# Data management needs assessment for the scale-up of district health information system and introduction of routine (essential) immunization module in Bauchi State, Nigeria, 2015

**DOI:** 10.11604/pamj.supp.2022.40.1.32458

**Published:** 2022-02-17

**Authors:** Olorunsogo Bidemi Adeoye, Oluwasegun Joel Adegoke, Chimeremma Nnadi, Hashim Elmousaad, Kunle Akerele, Patrick Nguku, Idowu Makinde, Richard Franka, Ndadilnasiya Endie Waziri

**Affiliations:** 1 African Field Epidemiology Network, Nigeria Country Office, Nigeria,; 2 Bauchi State Primary Health Care Management Board, Bauchi, Nigeria,; 3 Global Immunization Division, Center for Global Health, Centers for Disease Control and Prevention (CDC), Atlanta, Georgia

**Keywords:** Immunization data management, district health information system, routine immunization module, health management information system, health facility, local government, Bauchi State, Nigeria

## Abstract

**Introduction:**

the National Primary Health Care Development Agency, African Field Epidemiology Network, United States Centers for Disease Control and Prevention and the Bill and Melinda Gates Foundation are implementing a Routine Immunization (RI) Module as part of their Routine Health Data Management System based on the 2013 - 2015 Accountability Framework for RI in Nigeria. To inform planning and evidence-based decision making, a data management needs assessment was conducted in Bauchi state which was one of the states selected for the deployment of the DHIS2 RI module.

**Methods:**

desk reviews were conducted, and a semi-structured questionnaire was administered in four Local Government Areas (LGAs) in Bauchi state that were selected based on the initial evaluation of the performance of all 20 Bauchi LGAs. Ganjuwa and Shira were selected as high-performing LGAs and Alkaleri and Bogoro as low-performing LGAs. Four Health Facilities (HF) were selected in each LGA based on rural or urban classification, type of HFs (private or public), security and accessibility.

**Results:**

local Immunization Officers (LIOs) prepare monthly reports in high-performing LGAs, and Community Health Care workers are mostly (69%) responsible for report compilation at the HFs. Shira and Alkaleri met 77% and 44% of training indicator targets, respectively, in the previous 12 months. Data recording and reporting was the type of training received the most by health facility personnel. Functioning refrigerators were in all visited LGAs, working thermometer and updated temperature monitoring charts were available in all the cold chain stores. However, no health facility reported having available computers for data-related activities.

**Conclusion:**

this assessment provided an improved understanding of the Bauchi state Routine Health Data Management System and informed the content of the state-wide scale-up.

## Introduction

Immunization has been proven as one of the most cost-effective health interventions [1] and effective delivery of services is critical to induce an adequate population immunity to control vaccine-preventable diseases. Routine (Essential) Immunization (RI) services are provided in most countries as part of primary health care. Accurate immunization information is essential for immunization managers to track and improve programme performance and ultimately prevent morbidity and mortality [2,3]. Effective monitoring and evaluation of these immunization activities requires routine collection and analysis of a large amount of data [2,3].

In Nigeria, RI performance monitoring is achieved through a hierarchical administrative data monitoring system [4] where Health Care Workers (HCWs) at health facilities (HFs) aggregate monthly tallies of vaccination data using paper-based summary forms and report these data to health officers at the Local Government Area (LGA) level. The summary report is then entered on District Health Information System for all LGAs which then aggregates at the state and national level. These data are published as the states and country´s RI administrative data to estimate RI coverage [5,6]. Immunization programme managers at all levels depend on these coverage estimates to guide planning, review progress, and determine the areas that require intervention to mitigate low coverage and high drop-out rates [5].

The National Health Management Information System (NHMIS) coordinated by the Federal Ministry of Health (FMoH) uses the District Health Information System version 2 (DHIS2) to collect all routine health data in Nigeria. The National Primary Health Care Development Agency (NPHCDA) in collaboration with the African Field Epidemiology Network (AFENET), United States Centers for Disease Control and Prevention (CDC) and the Bill and Melinda Gates Foundation (BMGF) added an enhanced RI module component to the DHIS2 platform in 2014 under the 2013 - 2015 Accountability Framework for Routine Immunization in Nigeria [7,8].

DHIS2 was adopted by Nigeria National Council on Health as the singular platform for routine health data. Timely collection, collation, analysis, and appropriate reporting of quality data remain critical in the management of RI delivery systems. Problematic issues in data completeness, timeliness and quality have been identified on the NHMIS [9,10]. In response, the RI module functions within the NHMIS on the DHIS2 platform to capture and display key RI indicators on a customized dashboard in all 36 states and the Federal Capital Territory (FCT) [8,9] to identify and take to correct and avoid these issues in RI data.

Prior to roll out of the RI module in each state, a data management needs assessment is conducted to better inform planning and evidence-based decision-making. This assessment checks the availability of specific elements as relevant at the LGA and HF level, including appropriate data collection and reporting forms, infrastructure, human resource capacity and training needs.

We report on a data management needs assessment conducted in four LGAs and 16 HFs in Bauchi state in 2015 before RI DHIS2 implementation. The purpose of this assessment was to evaluate infrastructure and system capacity, the capacity of the HF and LGA RI teams for further workforce development, RI data access, use and quality, stock management data entry, data monitoring and analysis, as well as feedback and action taken on data analysis.

## Methods

### Study sites and design

Bauchi is in Northeastern part of Nigeria with a total estimated population of 4,653,066, infant <1 year of age target of 261,325 and under 5 years of age population of 1,306,624. There are 20 LGAs with 1,080 HFs, of which there are 822 public and 258 privates. The assessment used qualitative and quantitative methods. In Bauchi state, four LGAs were purposively selected. These selection criteria and scoring were: NHMIS completeness (≥95% scores 1; else 0), NHMIS timeliness (≥95% scores 1 else 0), coverage of BCG vaccine (≥85% scores 1; else 0), coverage of Measles-containing vaccine (≥85% scores 1; else 0), coverage of Pentavalent3 vaccine (third dose of diphtheria and tetanus toxoids and pertussis, hepatitis B and Haemophilus influenza type b vaccines) (≥85% scores 1; else 0) and Penta Drop-out rate (Penta1-Penta1) (<10% and ≥0 scores 1; else 0). A total score of 6 was possible; all 20 LGAs were ranked from highest to lowest. Ganjuwa and Shira were selected as high-performing LGAs and Alkaleri and Bogoro as representatives of low-performing LGAs based on these selection criteria. Four HFs were purposively selected in each LGA based on their rural or urban classification, type of HFs (private or public); and subjective assessment of representativeness and comparability. All needed to have appropriate security/accessibility.

### Advocacy visit to state level stakeholder/Training of field team

Prior to this assessment, we paid an advocacy visit to major state-level stakeholders comprised of State Ministry of Health staff including the immunization programme team, partners working in the state, the NPHCDA Zonal Coordinator, and the Polio Emergency Operations Center Operations Working Group. They were briefed about the plan, goals, and objectives of the assessment in preparation for the proposed DHIS2 scale-up and implementation of the customized RI module in Bauchi state.

### Cross-sectional study using interviewer-administered questionnaires

We conducted a cross-sectional assessment using interviewer-administered, semi-structured questionnaires. Four HFs were visited in each LGA. The respondents at HFs comprised (a) Health Facility Officer In-charge and (b) RI focal person at the HF level; while the respondents at the LGA level were (c) the Local Immunization Officer (LIO), (d) Cold Chain Officer (CCO), (e) Monitoring & Evaluation Officer (M&E) and (f) Director of Primary Health Care (DPHC). A total of five questionnaires (4 HFs and 1 LGA) were administered in each LGA.

### Data management

Questionnaire data were entered into Microsoft Excel spreadsheet version 2013 (Microsoft Corporation, Seattle, Washington). Some quantitative data collected during the interviews using Open Data Kit (ODK) in Comma Separated Value (CSV) format were analyzed on an ODK Aggregate server hosted by AFENET Nigeria.

### Data analysis

Prior to the commencement of data analysis, an analysis framework was developed using topics that were central to the objective of the assessment. Quantitative and Qualitative data analysis were conducted on the questionnaire responses. Excel was used to tabulate and calculate frequencies for each response that were categorical, and to create graphs from selected questionnaire data. For LGA level analysis, color codes were used to represent performance, where green color signifies meeting the performance indicator and red color signifies not meeting the performance indicator.

### Ethical consideration

The protocol for this assessment with interviews were covered under overall project approval granted by NPHCDA and FMoH. Prior to conducting the interviews, all participants provided verbal consent.

## Results

### LGA level assessment

Capacity building and workforce development: in the high-performing LGAs assessed (Ganjuwa and Shira), compiling and preparing monthly report is conducted by a team led by the LIO, while in low performing LGAs (Alkaleri and Bogoro), data compilation is conducted by the CCO alone. Only four (44%) and five (55%) out of 9 required training indicators were met during previous 12 months at LGA level in the high-performing LGAs, Alkaleri and Bogoro, respectively (Table 1). Seven (77%) and five (55%) training indicators were met in the low-performing LGAs, Shira and Ganjuwa, respectively.

**Table 1:** assessment of completion of at least one training received on routine immunization data in the previous 12 months

	Low performing LGAs*		High performing LGAs*	
	Alkaleri	Bogoro	Ganjuwa	Shira
Recording		1		1
Reporting			1	1
Entry	1		1	1
Quality	1		1	1
Dissemination		1	1	1
Analysis	1	1		1
Action Program improvement				1
NHMIS ¥	1	1		
DHIS2°		1		

Note: Green coloration signifies “Yes, training received” while red coloration signifies “No, training was not received”. ¥ National Health Management Information System (NHMIS)° District Health Information System (DHIS) (version 2) * High and low performance based on the six selection criteria explained under the study

***Routine immunization data access, use and quality:*** the Alkaleri LGA team reported having an updated RI micro-plan but was not able to present it upon request. Ganjuwa reported no usage of an RI micro-plan. On the other hand, Bogoro and Shira teams presented their micro-plans, most recently updated in 2015.

***Stock management data recording:*** written records of vaccine supply stocks using government-designed and -approved data collection tools were seen in Alkaleri, Bogoro and Shira. The Ganjuwa team gave a verbal response on tool availability; however, vaccine supply stocks were documented in an unofficial register (logbook). Functioning refrigerators were seen in all four LGAs; working thermometers and updated temperature monitoring charts were available at all the Cold Chain stores.

***Infrastructure and systems capacity:*** all four LGAs reported using computers for data-related activities; only Alkaleri, Bogoro and Ganujuwa LGAs reported entering data in the NHMIS through the DHIS 2 module. There is no existent capacity in Shira to enter data through the DHIS 2 module. Data entry activity was conducted by the M&E Officers of Bogoro LGA, while partner organizations oversee data entry in Alkaleri and Ganjuwa LGA. These three LGA teams reported that they spend approximately 4-6 days on data entry every month.

However, all three LGA teams entering data (Alkaleri, Bogoro and Ganjuwa) reported having problems with the ability to access and enter data in the NHMIS on DHIS2. Alkaleri and Ganjuwa reported specific problems like illegible writing on forms from the health facility limiting smooth data entry, while Bogoro reported having issues with internet modem access. There were intermittent electrical/connectivity problems in each LGA but Ganjuwa.

***Data review and analysis:*** Alkaleri, Ganjuwa and Shira LGAs reported District Vaccines and Devices Monitoring Tool (DVDMT) (the World Health Organization´s tool for vaccine and immunization monitoring) as the reference source for summarizing immunization data, while Bogoro reported using HMIS/DHHS2 as the reference source. Bogoro, Ganjuwa and Shira LGA teams reported that the LIO reviews records while Alkaleri reported that the M&E Officer reviews records.

***Feedback and action on data:*** all four LGA reported receiving feedback on the RI programme from the state in the previous six months. The major feedback received was on vaccine coverage and drop-out rates in the LGA. Only Shira mentioned receiving additional feedbacks on logistics. Feedback received monthly was given during regular meetings, letter report, telephone call and/or supervisory visit.

### Health facility level assessment

#### Capability building and workforce development

**Figure 1:**
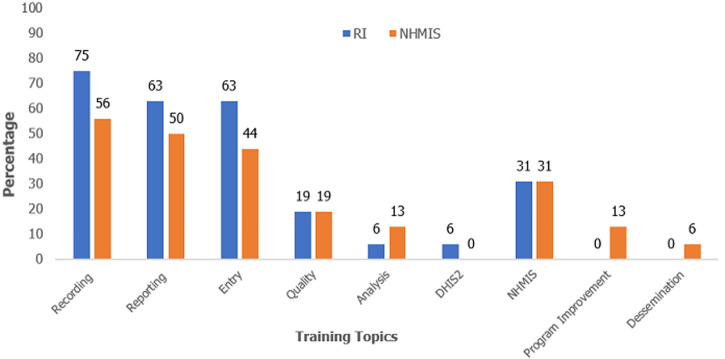
completion of training received on Routine Immunization (RI) data tools and National Health Management Information System (NHMIS) in the previous 12 months at Health facility (HF) level

Three (19%) of the 16 respondents reported that the HF officer in charge compiles and prepares monthly reports. In one (6%), it was done by the RI focal person, in one (6%) by the M&E officer and in 11 (69%) by the community health worker (CHW). An assistant is available in four (25%) of the HFs to fill-out forms during immunization sessions. In the responses of all HFs (100%), training was received on recording and reporting RI data in the previous 12 months (Figure 1).

### Routine immunization data access, use and quality

In the 16 facilities visited, one (6%) had no RI micro-plan; three (19%) verbally reported having a micro-plan and 12 (75%) reported that they had their micro-plan on-hand (physically sighted) (nine of which were updated within the previous 12 months). Four HFs (25%) verbally reported that their micro-plan was updated. The reported sources of NHMIS and RI Data Collection Tools were LGA immunization programmes for five (31%) HFs, state PHC for 11 (56%) and LGA PHCs for two (13%).

All the 16 responding HFs stated that programme reports are sent monthly to the LGA. Eleven (56%) of HFs reported that they send their monthly report on a specific day within the month. One HF (6%) that reports on the last day of the month, and one (6%) report in the first week of every month; three (31%) HFs report on an irregular basis.

### Stock management data recording

Thirteen (81%) of the visited facilities could present a written current stock of vaccine. Functioning refrigerators were reported in six (38%) of HFs; only five (31%) reported having a working thermometer in each refrigerator currently storing vaccine and four (25%) having a temperature-monitoring chart. All health facilities reported having intermittent power supply.

### Infrastructure and system capacity

No HF reported having computers for data-related activities; 15 (94%) reported having mobile phone connectivity despite no internet connectivity. The availability of vehicles for transportation of reports to LGA was reported in five (31%) of the visited facilities, all of which were public HFs. Fifteen (94%) of HFs reported year-round accessibility for supportive supervision and vaccine delivery. Three (19%) facilities reported that the driving time to the LGA office is 30-60 min while thirteen (81%) reported less than 30 min to the LGA office.

### Data use analysis

**Figure 2:**
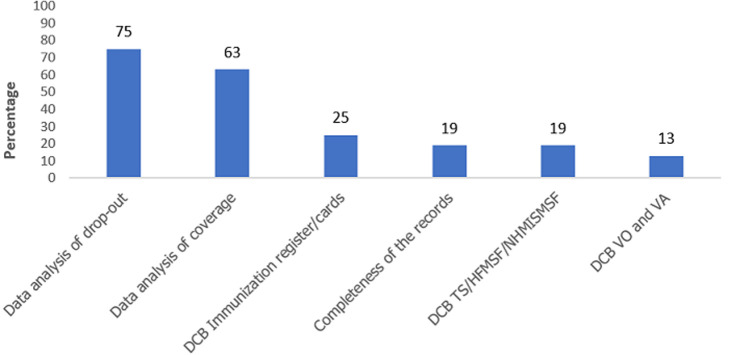
types of analysis conducted monthly in the 16 selected health facilities

**Figure 3:**
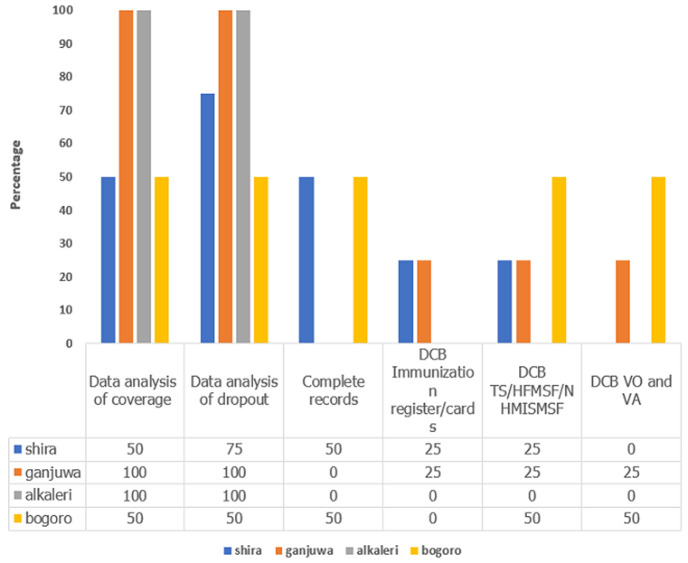
types of analysis conducted monthly in the 16 assessed health facilities

Record and report review at the HF level is being done by HF officer in charge in 15 (94%) of facilities visited. Record review was performed in 15 (94%) public HFs visited and 100% of private facilities. Twelve (72%) HFs reported that the most common analysis conducted in the facilities is Penta dropout on the monthly summary sheets (Figure 2, Figure 3).

### Feedback and action data

Nine (56%) HFS reported receiving feedbacks from the LGA on routine immunization data in the previous 6 months; the most common feedback was regarding vaccine coverage in six (40%) HFs and drop-out rate in five (31%).

## Discussion

The assessment conducted at the LGA, and HF levels revealed several issues regarding the level of readiness of the Bauchi state health management system for the full-state introduction of the customized RI module on the DHIS2 platform.

This assessment confirmed that Ganjuwa and Shira LGAs were indeed high-performing areas. LIOs in these high-performing LGAs were responsible for monthly data reporting in coordination with other LGA team members. In contrast, the CCO in Alkaleri and Bogoro is responsible for monthly reporting. This capacity gap in role identified will be an input to the advocacy and planning of state-wide expansion. CHWs played a substantial role in ensuring effective data management at the HFs. The Bauchi state PHC needs to heavily consider the inclusion of CHWs in subsequent capacity building and engagement sessions. This assessment reveals that their involvement appeared, as they are responsible for 69% of monthly report compilation and preparation.

The Reach Every Ward (REW) Micro-plan is a document that helps organize immunization services at HFs and communities and its one of the datasets reported monthly on the Nigeria DHIS2 platform. This assessment revealed that only about 50% of the LGAs and HFs had updated micro-plans the in previous 12 months. The micro-plan datasets contain some data elements that serve as denominators for some Indicators on DHIS2 and if not available may result in falsely low or falsely high-performance indicators. It is therefore necessary to include REW Microplanning reporting and its importance in implementing RI in the training curriculum before statewide implementation.

Data entry unto the DHIS2 platform and transmission is conducted at the LGA level; this necessitates a good road network that reduces the travel time for submission of reports from the HFs to the LGA, a functioning computer system, internet connectivity, and trained personnel with data entry skills. The varying infrastructure issues reported here indicate a needed baseline for individual HFs and LGAs and should become an advocacy point for improved NHMIS use and quality in the state. The presence of dedicated, trained, or trainable officers at the LGA and HF level would be of a great advantage to the state HMIS and would promote ownership and sustainability, which is part of what the proposed scale-up and introduction project intends to achieve.

The results of this assessment concur with some results of the Nigeria Health Sector Assessment (NHSA) (phase 2) [11] in March 2015 by the United Nations Foundation (UNF). Our assessment demonstrated improvement over most of the results reported by UNF assessment. The UNF assessment established that the primary source of power is a national or community grid, however, frequent, or prolonged power interruptions are common. Fifty percent (50%) of LGA M&E officers are trained on DHIS2. Data on DHIS2 training at the state level was not available. Sufficient training (i.e., statistics, software and database maintenance, and epidemiology) has taken place for health Information Communication Technology (ICT) staff at the MOH in the previous year but was dependent on donor/external funding. HMIS training has taken place at 2 of 3 LGAs in the previous 6 months, and 29% of facilities reported that Health ICT related training activities (i.e., data collection, self-assessment, analysis, and presentation) have taken place in the previous one year, provided by the government. All state and LGA health offices have a designated health information officer.

The assessment serves as a quick appraisal of the RI data system in the selected LGAs of the state and not the entire NHMIS data system in the state, therefore interpretation/generalization of the data should be made with caution. Two HFs were not assessed due to the unavailability of the Health Care Workers.

## Conclusion

This assessment provided an additional understanding of the Bauchi state Routine Health Data Management System and guidance on best practices and areas of improvements to be incorporated into the proposed DHIS2 scale up project in the state.
